# Microscopic Study on Excitation and Emission Enhancement by the Plasmon Mode on a Plasmonic Chip

**DOI:** 10.3390/s20226415

**Published:** 2020-11-10

**Authors:** Hinako Chida, Keiko Tawa

**Affiliations:** School of Science and Technology, Kwansei Gakuin University, 2-1 Gakuen, Sanda, Hyōgo 669-1337, Japan; h.chida@kwansei.ac.jp

**Keywords:** plasmon mode, microspectroscopy, surface plasmon resonance, fluorescence microscopy, plasmon enhancement

## Abstract

Excitation and emission enhancement by using the plasmon mode formed on a plasmonic chip was studied with a microscope and micro-spectroscope. Surface plasmon resonance wavelengths were observed on one-dimensional (1D) and two-dimensional (2D) plasmonic chips by measuring reflection and transmission spectra, and they were assigned to the plasmon modes predicted by the theoretical resonance wavelengths. The excitation and emission enhancements were evaluated using the fluorescence intensity of yellow–green fluorescence particles. The 2D grating had plasmon modes of $k_{gx45}{}^{\left(2\right)}$ (diagonal direction with m = 2) in addition to the fundamental mode of $k_{gx}{}^{\left(1\right)}$ (direction of a square one side) in the visible range. In epifluorescence detection, the excitation enhancement factors of $k_{gx}{}^{\left(2\right)}$ on the 1D and 2D chips were found to be 1.3–1.4, and the emission enhancement factor of $k_{gx45}{}^{\left(2\right)}$ on the 2D chip was 1.5–1.8, although the emission enhancement was not found on the 1D chip. Moreover, enhancement factors for the other fluorophores were also studied. The emission enhancement factor of $k_{gx}{}^{\left(1\right)}$ was shown to depend on the fluorescence quantum yield. The emission enhancement of 2D was 1.3-fold larger than that of 1D considering all azimuth components, and the 2D pattern was shown to be advantageous for bright fluorescence microscopic observation.

## 1. Introduction

Recently, surface plasmon resonance (SPR) has been applied to biosensors [1,2,3,4,5] and bioimaging [6,7,8,9,10]. In particular, propagated SPR using prisms or diffraction grating has been shown to enhance the electric field more widely than localized SPR. The optical system required for the plasmonic chip—grating-coupled SPR (GC-SPR) [11,12,13,14,15,16,17] using diffraction grating—is simpler than the prism-coupled SPR, and the electric field enhancement of the excitation field can be controlled by the pitch of the grating or the incident angle and wavelength.

The resonance condition of GC-SPR is shown in Equation (1) [11]. The electric field enhancement occurs at resonance conditions on the chip.

(1) $$k_{spp}=k_{phx}\pm mk_{g}\left(m=0,1,2,\ldots \right)$$

(2) $$2\pi /\lambda \sqrt{\varepsilon_{d}\varepsilon_{m}/\left(\varepsilon_{d}+\varepsilon_{m}\right)}=n\left(2\pi /\lambda \right)\sin\theta \pm m\left(2\pi /\Lambda \right)$$

(3) $$\sqrt{\varepsilon_{d}\varepsilon_{m}/\left(\varepsilon_{d}+\varepsilon_{m}\right)}/\lambda =\pm m/\Lambda \;\;\;\;\;\;\;\;\;\;\;\;\;\left(at\;\theta =0\right)$$

where $k_{spp},\;k_{phx}\;\mathrm{and}\;k_{g}$ denote the wavenumber vector of the surface plasmon polariton, the incident light in x direction and the grating, respectively, and m is the integer showing the order of the plasmon mode. In Equations (2) and (3), $\varepsilon_{d}\;\mathrm{and}\;\varepsilon_{m}$ are the complex dielectric constants of the dielectric and metal, respectively. n, θ and Λ denote the refractive index of dielectric media, the incident angle and the pitch of the grating.

**Figure 1:**
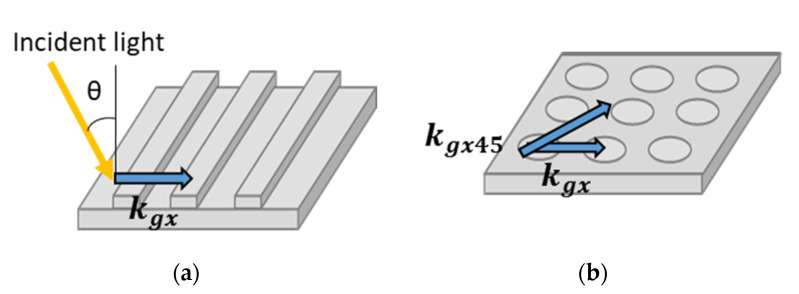
Grating vectors of (**a**) a one-dimensional (1D) plasmonic chip and (**b**) a two-dimensional (2D) plasmonic chip. $k_{gx}$ and $k_{gx45}$ are grating vectors coupled with the lateral and diagonal direction of pitch.

Under vertical incidence—i.e., θ = 0—Equation (2) is transformed into Equation (3). As shown in Figure 1a, a one-dimensional (1D) grating (line and space) plasmonic chip shows one kind of plasmon mode with a grating vector of the order of *m*-th, $k_{gx}{}^{\left(m\right)}$, for a one-sided direction, while a two-dimensional (2D) grating (hole array) plasmonic chip has two kinds of plasmon modes with grating vectors of the order of *m*-th, $k_{gx}{}^{\left(m\right)}$ and $k_{gx45}{}^{\left(m\right)}$ for a diagonal direction (Figure 1b).

The surface plasmon-field enhanced fluorescence (SPF) method can detect analytes with high sensitivity because of the enhanced electric field of excitation light and the emission enhancement based on the surface plasmon coupled emission (SPCE) [18,19,20,21]. In our laboratory, the bright fluorescence images of breast cancer cells [22,23] and neuron cells [10,24,25,26] cultured on the plasmonic chip have been observed using fluorescence microscopy [27] due to the product of the excitation and emission enhancement effects [20]. However, the fluorescence enhancement observed in these images has not been precisely assigned to the plasmon modes.

**Figure 2:**
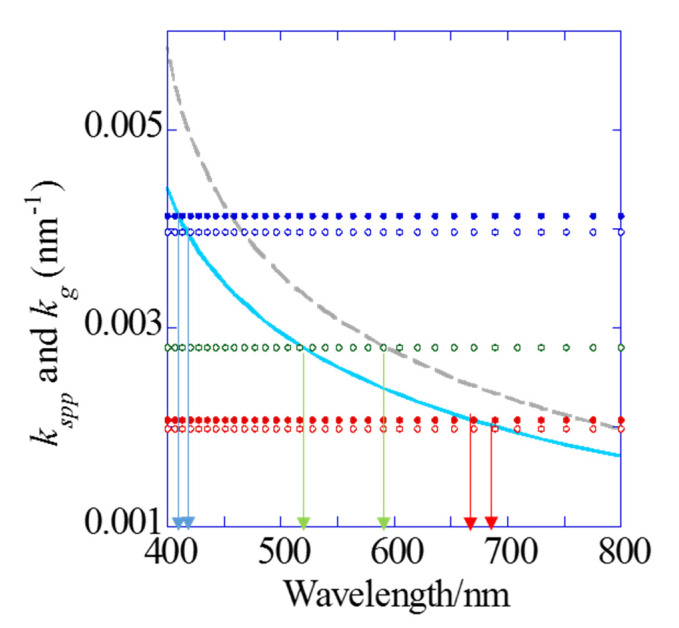
Theoretical dispersion curves of wavenumber vectors $k_{spp}$ for the silver/water (blue solid line) and silver/resin interfaces (gray broken line), and grating vectors $k_{gx}{}^{\left(1\right)}$ and $k_{gx}{}^{\left(2\right)}$ for a 480 nm pitch on the 1D chip (red and blue dotted lines (closed circles)), $k_{gx}{}^{\left(1\right)}$, $k_{gx}{}^{\left(2\right)}$ and $k_{gx45}{}^{\left(2\right)}$ for a 500 nm pitch on the 2D chip (red, blue and green dotted line (open circles), respectively).

In this study, the resonance wavelength was investigated from experiments and theory. In the reflection system, the resonance at the interface between a metal layer and water is mainly considered. For a 1D chip with Λ = 480 nm, $k_{gx}{}^{\left(m\right)}s$ of m = 1 and 2 can be observed in visible range. On the other hand, for a 2D chip with Λ = 500 nm, $k_{gx}{}^{\left(m\right)}s\;\mathrm{of}$ m = 1 and 2 and a $k_{gx45}{}^{\left(m\right)}\;\mathrm{of}$ m = 2 can be observed. In the transmitted light observation, the resonance at the interface between the metal layer and UV-curable resin on the rear panel of a chip should be also considered. Therefore, on the rear panel, the 1D grating additionally shows one kind of plasmon resonance with a grating vector, $k_{gx}{{}^{\left(2\right)}}_{back}$, and the 2D grating plasmonic chip shows two kinds of grating vector, $k_{gx}{{}^{\left(2\right)}}_{back}$ and $k_{gx45}{{}^{\left(2\right)}}_{back}$. These grating vectors coupled with the plasmon—called the plasmon mode—can theoretically fix the resonance wavelength, as shown in Figure 2 and Table 1.

In this study, the resonance wavelengths of the 1D and 2D chips were measured at the silver/water interface of the top panel and silver/resin interface of the rear panel by microspectroscopy under both epi-illumination and transmitted-light optical systems, and they were individually assigned to the plasmon modes predicted from resonance theory. We clarified which plasmon mode, i.e., excitation or emission enhancement, contributed to the total fluorescence enhancement of the fluorescent nanoparticles (YG). Further, the emission enhancement was evaluated for three kinds of fluorophores, and the difference in the plasmon enhancement effect was also discussed.

## 2. Materials and Methods

### 2.1. Fabrication of 1D and 2D Plasmonic Chips

**Figure 3:**
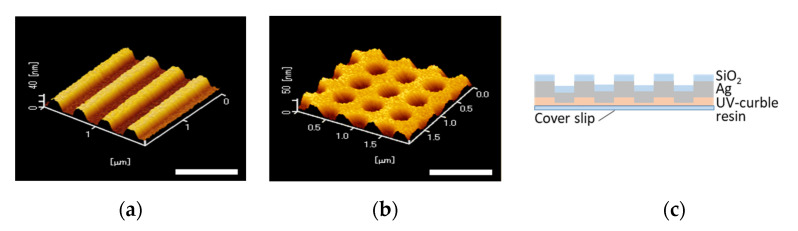
Atomic force microscopy (AFM) image of (**a**) 1D and (**b**) 2D plasmonic chips, and (**c**) the cross section view of the plasmonic chip. Bar corresponds to 1 µm.

Replicas of 1D and 2D periodic structures were prepared by the UV nanoimprint method. A photocurable resin (PAK02-A; TOYO GOSEI) was dropped on the cover glass and covered with a mold, and then exposed with UV light. Then, Ti, Ag, Ti and SiO_2_ were deposited on the replica by the radio frequency (Rf)-sputtering method, and the film thickness of thin silver and silica layers were 40 ± 10 nm [28] and 20 ± 10 nm [29,30], respectively. The grating pattern was a 4 mm square located in the center of a 25 mm square chip. The structure of the plasmonic chip was measured by an atomic force microscope (AFM), as shown in Figure 3. The pitches of the 1D and 2D periodic structures were 484 nm and 504 nm, respectively. Furthermore, the depth and width of the grooves were 30 nm and around a half of pitch for both replica.

### 2.2. Sample Preparation

A one percent aqueous solution of (3-aminopropyl) triethoxysilane (APTES) was dropped on the chip for modification of SiO_2_ surface. After 1 h, it was washed with milli-Q water. For the microscopic observation, after cover glass was attached to the double-sided tape pasted on both edges of a plasmonic chip, sample solution was injected into the space between the cover glass and the chip. In the fluorescence observation of Cy5 streptavidin conjugate (Cy5-SA; Biolegend, San Diego, CA, USA) [31] and Qdot streptavidin conjugates (Qdot655-SA; Thermo Fisher Scientific, Tokyo, Japan) [32], biotin-poly(ethylene glycol)-carbonate-(*N*-hydroxysuccinimide) (biotin-PEG-COO-NHS, NOF America Corporation, BRIGHT BI-050TS) aqua solution prepared at 2 mM was further modified to an amino group on the silica surface. Then, 20 µL of solution with streptavidin-conjugated fluorescent compounds was injected and after incubation for 10 min, it was rinsed with 60 µL of phosphate-buffered saline (PBS) solution. On the other hand, carboxylate-modified microspheres with a diameter of 0.02 μm, yellow–green fluorophore (YG; Thermo Fisher Scientific, Tokyo, Japan) [33] were injected onto the plasmonic chip surface modified with APTES in the microscopic observation. Three types of fluorophores were prepared at the following concentrations summarized in Table 2.

### 2.3. Microscopic Measurement

An upright-inverted microscope (BX51WI and IX71, special-made, Olympus, Tokyo, Japan) mounting a spectrometer switchable to imaging (CLP-50, Andor), an electron multiplying charge-coupled device camera (EM-CCD, Luca-R, Andor), and a mercury lamp (on an upright side), a xenon lamp (on an inverted side) and halogen lamps (on both sides) was used. Two kinds of objective lenses (NA = 0.08 and 0.06, on upright and inverted side, respectively) and a bright field filter attached to both sides were used for measuring reflection and transmission spectra, which were taken in the wavelength range of 400–750 nm at the water interface. The ratio of the light intensity in the grating structure to that in the flat metal area corresponds to the relative reflection or relative transmission spectra of a plasmon field to those of flat metal area.

The fluorescence filters used for fluorescence imaging and spectra are summarized in Table 3. Fluorescence spectra of YG were measured in the range of 550 to 750 nm with a cyan fluorescent protein (CFP) excitation (exCFP (425–445 nm)) filter. The exposure time and EM gain were adjusted so that the brightness was 12,000 counts. The enhancement factor spectra corresponded to the ratio of fluorescence intensities between the inside and outside of a grating pattern; i.e., the fluorescence intensity in the grating pattern divided by that in the flat metal area. The total enhancement factors were calculated by integrating the fluorescence enhancement over the fixed wavelength range of 660–710 nm. Fluorescence images were measured with an exCFP excitation filter for excitation and with a green fluorescent protein (GFP) emission (emGFP (495–540 nm)) filter or a Cy3 emission (emCy3 (570–6155 nm)) filter. The fluorescence enhancement was evaluated by the ratio of fluorescence intensity in the region of interest (ROI) for grating area to that for flat metal area. Fluorescence images were taken at four corners of the grating pattern of the chip in order to statistically evaluate the enhancement factors. In the experiment of fluorophore dependence on the enhancement factors, the fluorescent images of the three samples were measured using with exCFP (425–445 nm), exGFP (495–540 nm), exCy3 (510–555 nm) and exCy5 (600–650 nm) filters for excitation and with emCy5 (670~715 nm) filter for emission.

## 3. Results and Discussion

### 3.1. Resonance Wavelength Indicated by Reflection and Transmission Spectra

**Figure 4:**
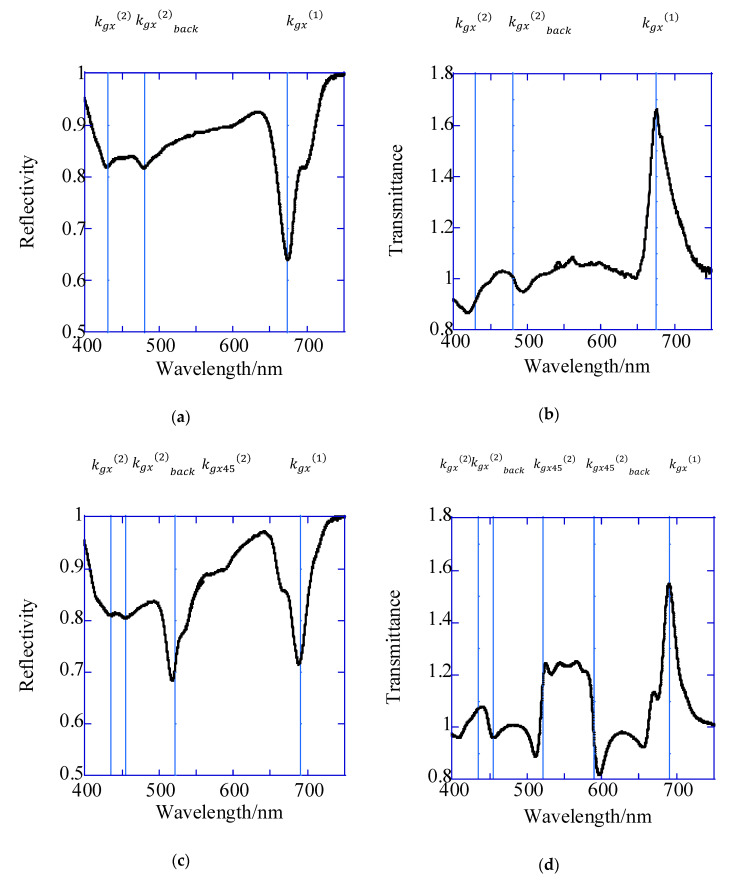
Relative reflection (**a**,**c**) and relative transmission (**b**,**d**) spectra for 1D (**a**,**b**) and 2D (**c**,**d**) plasmonic chips.

The relative reflection and transmission spectra measured for 1D and 2D plasmonic chips for the water interface and the wavelengths of plasmon modes calculated from resonance theory (blue solid lines) are shown in Figure 4. In the transmitted-light observation (Figure 4b,d), the plasmon resonances were considered to be formed on both panels of a chip; i.e., the back and the top. In the reflection spectra for the 1D plasmonic chip, resonance dips were observed at 430 nm, 480 nm and 672 nm. Although they were shifted to a 2–20 nm longer wavelength than those described in the resonance theory shown in Table 1, they were assigned to the plasmon modes of $k_{gx}{}^{\left(2\right)}$, $k_{gx}{{}^{\left(2\right)}}_{back}$ and $k_{gx}{}^{\left(1\right)}$, respectively (Figure 4a). On the other hand, in the transmitted-light observation, the inflection points were also observed at 430 nm and 480 nm which were assigned to the plasmon modes of $k_{gx}{}^{\left(2\right)}$ and $k_{gx}{{}^{\left(2\right)}}_{back}$. In addition to these, the peak observed at 672 nm was assigned to be $k_{gx}{}^{\left(1\right)}$ in the reflection spectra (Figure 4b).

In the reflection spectra for the 2D plasmonic chip, resonance wavelengths of 435 nm, 455 nm, 522 nm and 693 nm were obtained. They were also shifted to a 2–20 nm longer wavelength and were assigned to plasmon modes of $k_{gx}{}^{\left(2\right)}$,$\;k_{gx}{{}^{\left(2\right)}}_{back},k_{gx45}{}^{\left(2\right)}$ and $k_{gx}{}^{\left(1\right)}$, respectively (Figure 4c). In transmitted-light observation for the 2D chip, the inflection points were also observed at 435 nm, 455 nm and 522 nm, and the peak was at 695 nm, which was assigned to the modes of $k_{gx}{}^{\left(2\right)}$, $k_{gx}{{}^{\left(2\right)}}_{back}$ and $k_{gx}{}^{\left(1\right)}$. Additionally, the inflection points were observed at 594 nm; these were assigned to the mode of $k_{gx45}{{}^{\left(2\right)}}_{back}$ (Figure 4d).

### 3.2. The Fluorescence Spectra and Fluorescence Image of YG

**Figure 5:**
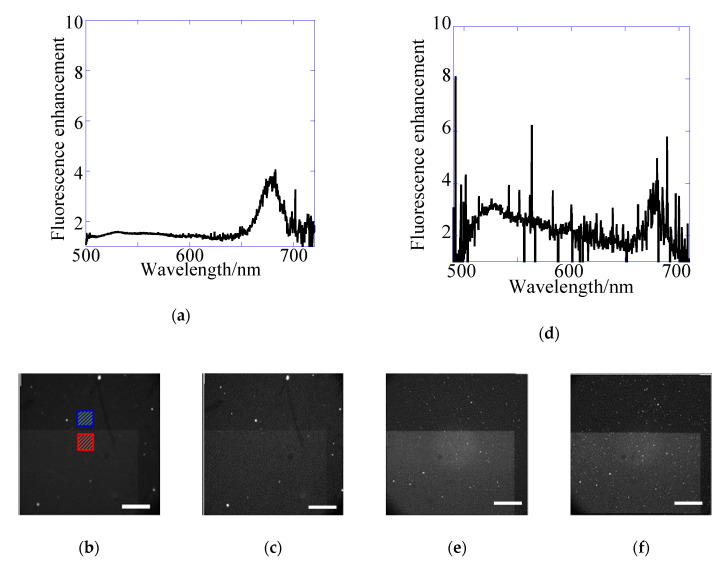
(**a**,**d**) fluorescence spectra and images of yellow–green (YG) fluorescence detected with (**b**,**e**) emGFP (495–540 nm) and (**c**,**f**) emCy3 (570–615 nm) filters for the 1D plasmonic chip on epi-fluorescence (**a**–**c**) and transmitted-light systems (**d**–**f**). The fluorescence enhancement was evaluated by the ratio of the fluorescence intensity in region of interest (ROI) for the grating area (red square) to that for flat metal area (blue square) in fluorescence images as shown in (**b**).

To evaluate the total enhancement factor and to distinguish the enhancement effect of excitation from that of emission, the emission spectra of YG were measured at first on the 1D chip in the wavelength range of 500–700 nm by using the CFP excitation filter (Figure 5a,d). The fluorescent particle YG is useful for the study of the plasmon mode because of the broad absorption and fluorescence spectra. Total fluorescence enhancement factor is composed of the product of excitation enhancement, Ex and emission enhancement, Em. Therefore, Em can be evaluated by using the experimental value of total enhancement factor obtained under the excitation not involving plasmon modes in the wavelength range of excitation filter (Ex = 1). On the other hand, Ex can be evaluated by total enhancement factor obtained under the detection not involving plasmon modes in the wavelength range of emission filter (Em = 1). The exCFP filter (425–445 nm) includes resonance wavelength of 430 nm for $k_{gx}{}^{\left(2\right)}$. In the epi-fluorescence imaging, the total enhancement factors evaluated using ROI as depicted in Figure 5b increased up to 1.4-fold with the GFP emission filter (emGFP; 495–540 nm) and Cy3 emission filter (emCy3; 570–615 nm), which were used as the condition not involving plasmon modes in the emission range, i.e., Em = 1. Therefore, the excitation enhancement of $k_{gx}{}^{\left(2\right)}$ was determined as a 1.4-fold increase (Figure 5a–c).

In transmitted-light illumination, the emGFP filter (495–540 nm) and Cy3 emission filter (emCy3; 570–615 nm) were also used as the condition without plasmon mode in the emission range. As shown in Figure 4a,b, spectra for $k_{gx}{}^{\left(2\right)}\;\mathrm{and}$ $k_{gx}{{}^{\left(2\right)}}_{back}$ of plasmon modes were broadly extended. Therefore, the back mode ($k_{gx}{{}^{\left(2\right)}}_{back}$) could contribute to the excitation enhancement in the transmitted-light system, although this was not found in the epi-system. The total enhancements were 2.0–2.5-fold, and the excitation enhancement by both $k_{gx}{}^{\left(2\right)}$ and $k_{gx}{{}^{\left(2\right)}}_{back}$ was also found to be a 2.0–2.5-fold increase in the transmitted-system (Figure 5d–f). The values were larger than those observed for epi-mode because of the contribution by both plasmon modes (top and back sides), in spite of the large noise due to the small fluorescence intensity under the back illumination (Table 4 and Table 5). All total enhancement factors evaluated from the spectra include 10% coefficient variation (CV).

**Figure 6:**
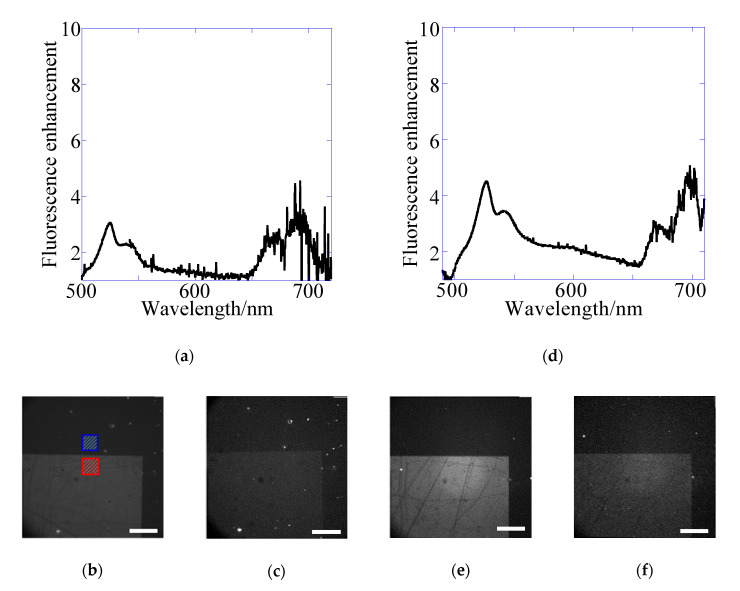
(**a**,**d**) fluorescence spectra and images of YG detected with (**b**,**e**) emGFP (495–540 nm), (**c**,**f**) emCy3 (570–615 nm) filters for the 2D plasmonic chip on epi-fluorescence (**a**–**c**) and transmitted-light systems (**d**–**f**). The fluorescence enhancement was evaluated by the ratio of the fluorescence intensity in ROI for the grating area (red square) to that for flat metal area (blue square) in fluorescence images as shown in (**b**).

Figure 6 and Table 6 and Table 7 show the result of YG for the 2D chip. In the epi-fluorescence observation, emCy3 (570–615 nm) was used for the emission side under the no plasmon mode. The results showed that the excitation enhancement of $k_{gx}{}^{\left(2\right)}$ was evaluated as 1.3-fold. This value was the same as that evaluated for the 1D chip. Regarding the emission detected at the emGFP (495–540 nm) filter, resonance wavelength by $k_{gx45}{}^{\left(2\right)}$ was included in the range of emGFP filter. Therefore, the emission enhancement due to $k_{gx45}{}^{\left(2\right)}$ was evaluated as a 1.5–1.8-fold increase for both results from the spectra (Figure 6a) and images (Figure 6b).

In the transmitted-light observation at the emCy3 (570–615 nm) filter, the emission enhancement effect was not included, i.e., Em = 1.0-fold. Total enhancement factors of 2.1–2.2 were based on the excitation enhancement due to $k_{gx}{}^{\left(2\right)}\;\mathrm{and}\;k_{gx}{{}^{\left(2\right)}}_{back}$ plasmon modes, which were almost the same as those evaluated for the 1D chip. In detection with emGFP (495–540 nm), the emission enhancement due to $k_{gx45}{}^{\left(2\right)}$ was added. The total enhancement was 2.8–3.4-fold, and therefore, the emission enhancement of $k_{gx45}{}^{\left(2\right)}$ was evaluated as 1.3–1.5-fold (Table 7). These values were almost consistent with Em of 1.5–1.8-fold in epifluorescence (Table 6). All enhancement factors evaluated from spectra include 10%–20% CV.

### 3.3. Fluorophore Dependence on the Enhancement Factors

The fluorophore dependence on the enhancement factors at the water interface was studied in a wide wavelength range on the 1D and 2D chips. Under the fixed emission wavelength, the excitation wavelength was changed from resonance to off-resonance fields, and the enhancement factors were evaluated for each plasmon mode. The standard deviation for the fluorescence intensities in their spectra was larger; i.e., the CV of enhancement factors was larger than that obtained in fluorescence images. Therefore, the enhancement factors evaluated in fluorescence images are summarized in Table 8.

In the case of excitation with exCFP (425–445 nm), exGFP (460~480 nm), exCy3 (510~555 nm) and exCy5 (600~650 nm), the fluorescence was detected with emCy5 (670~710 nm). As denoted in Table 8 and Table 9, some instances were not properly measured because the extinction coefficient for the fluorophore is too small in the excitation wavelength range. In particular, the range of emCy5 corresponds to the fluorescence tail for YG. For YG excited with exGFP (460–480 nm) and exCy3 (510–555 nm), the emission enhancement factor by the plasmon mode of $\;k_{gx}{}^{\left(1\right)}$ was decided as 3.1–3.2-fold. For Cy5-SA excited with exCy3 (510–555 nm) and exCy5 (600–650 nm), the emission enhancement factor by the plasmon mode of $k_{gx}{}^{\left(1\right)}$ was determined to be 5.3–5.6-fold. On the other hand, for Qd655-SA excited with exGFP (460–480 nm), exCy3 (510–555 nm) and exCy5 (600–650 nm), the emission enhancement factor by the plasmon mode of $k_{gx}{}^{\left(1\right)}$ was determined as 2.1–2.2-fold. The mean value was 2.2. 

Therefore, under excitation with exCFP (425–445 nm), the emission enhancement by $k_{gx}{}^{\left(1\right)}$ was considered to be the same value—2.1-fold—and the excitation enhancement based on $k_{gx}{}^{\left(2\right)}$ was evaluated as 1.2-fold. This is consistent with the results—i.e., 1.4-fold—under exCFP and emGFP (or emCy3) found in Table 4.

These results suggest that emission enhancement has a relationship to the fluorescence quantum yield (Table 2); i.e., the smaller the quantum yield of fluorophores, the larger plasmon enhancement.

Enhancement factors for fluorophores were similarly analyzed in the 2D chip. For YG excited with exGFP (460–480 nm), the emission enhancement by $k_{gx}{}^{\left(1\right)}$ was evaluated as 4.0. Compared with the 1D chip (3.1-fold), the emission enhancement by $k_{gx}{}^{\left(1\right)}$ was 1.3-fold in the 2D chip. The difference in the structure of 1D and 2D chips provided further emission enhancement, and the 2D structure was found to increase the coupling rate between the fluorescence from fluorophores and the plasmon mode. Such an enhancement due to the 2D chip was not observed in the excitation enhancement, as seen in Table 4 and Table 6. For YG excited with exCy3 (510–555 nm), the total enhancement was 5.2-fold (Table 9). The excitation enhancement effect of $k_{gx45}{}^{\left(2\right)}$ was determined to be 1.3-fold using the emission enhancement factor by $k_{gx}$^(1)^ of 4.0.

For Cy5-SA excited with exCy5 (600–650 nm) without excitation enhancement, the emission enhancement by $k_{gx}$^(1)^ was determined to be 7.5-fold (Table 9). Using 7.5-fold emission enhancement, the excitation enhancement by $k_{gx45}$^(2)^ was evaluated to be 1.3-fold under the excitation with exCy3 (510–555 nm). This is consistent with the value obtained for excitation enhancement due to $k_{gx45}$^(2)^ for YG.

For QD655-SA excited with exGFP (460–480 nm) and exCy5 (600–650 nm) under no excitation enhancement, the mean emission enhancement by $k_{gx}$^(1)^ was 2.9-fold (Table 9). Using 2.9-fold, the excitation enhancement by $k_{gx45}$^(2)^ was evaluated to be 1.3-fold under excitation with exCy3 (510–555 nm) as well as Cy5-SA. As described above, the excitation enhancement factor by $k_{gx45}$^(2)^ is 1.3 regardless of fluorophores. In the excitation with exCFP (425–445 nm), the emission enhancement by $k_{gx}{}^{\left(1\right)}$ was considered to be 2.8-fold, and therefore the excitation enhancement due to $k_{gx}{}^{\left(2\right)}$ was evaluated as 1.1-fold. This is almost consistent with the results of Ex (excitation enhancement) values of 1.3-fold under the exCFP and emGFP (or emCy3) conditions, as seen in Table 6.

The emission enhancement by $k_{gx}$^(1)^ in YG, Cy5-SA and Qdot655-SA could be evaluated as 4.0, 7.5 and 2.8-fold, respectively. These values were consistently 1.3-fold larger than the those for the 1D chip (3.2, 5.6 and 2.1).

The larger enhancement in the 2D chip is considered to be due to the emission enhancement contributed by the azimuth component, because the 2D chip has higher symmetry, and fluorescence from fluorophores can effectively couple with plasmon modes considering all the azimuth angles (360°) under a microscope. In brief, the azimuth angle range coupling between $k_{gx}{}^{\left(1\right)}$ of the 1D chip and fluorescence is 0–90°, but the angle between $k_{gx}{}^{\left(1\right)}$ of the 2D chip and fluorescence is 0–45°. Therefore, the 2D plasmonic chip can provide a higher coupling rate with plasmon than the 1D, and the emission enhancement in the 2D chip is considered to be improved.

## 4. Conclusions

In the observation of reflection and transmission spectra, the resonance wavelength of 1D and 2D plasmonic chips for silver/water and silver/resin interfaces were assigned to the plasmon modes coupled with grating vectors, which were calculated from theoretical resonance conditions. In the fluorescence spectra and fluorescence images of YG, the excitation enhancement of $k_{gx}{}^{\left(2\right)}$ in the 1D and 2D chip were almost the same as 1.3–1.4-fold, and the difference in the structure between 1D and 2D chips did not improve the excitation enhancement. However, in the excitation with exCy3, the additional vector, $k_{gx45}{}^{\left(2\right)}$, in the 2D chip improved the excitation enhancement up to 1.3-fold larger than that in 1D chip. On the other hand, the emission enhancements with $k_{gx}{}^{\left(1\right)}$ of YG, Cy5-SA and Qdot655-SA were evaluated as 3.2, 5.5 and 2.2-fold in the 1D plasmonic chip and 4.0, 7.5 and 2.9-fold in the 2D plasmonic chip, respectively. These results were consistent with the order of the fluorescence quantum yield. The emission enhancement of $k_{gx}{}^{\left(1\right)}$ in the 2D plasmonic chip with any fluorophores as about 1.3-fold larger than that of the 1D plasmonic chip because of the azimuth component.

Therefore, it is clear that emission detection on a 2D chip can provide a brighter emission image than a 1D chip in a wide wavelength range, due to the effective utilization of azimuth components and an additional grating vector. The 2D plasmonic chip shows an advantage in fluorescence microscopic imaging. Moreover, a greater enhancement was expected by using fluorophores with a low fluorescence quantum yield that could make effective use of plasmon modes. The plasmonic chip can overcome the darkness in fluorescence images for fluorophore with low fluorescence quantum yield.

## Figures and Tables

**Table 1:** The plasmon resonance wavelength of a plasmon mode predicted theoretically at silver/water and silver/resin interfaces on 1D and 2D chips.

Interface	Resonance Wavelengths and Plasmon Modes	
	Silver/Water (Top Side)	Silver/Resin (Rear Side)
1D chip	410 nm$:\;k_{gx}{}^{\left(2\right)}$ 670 nm: $k_{gx}{}^{\left(1\right)}$,	460 nm: $k_{gx}{{}^{\left(2\right)}}_{back}$
2D chip	415 nm: $k_{gx}{}^{\left(2\right)}$ 520 nm: $k_{gx45}{}^{\left(2\right)}$ 695 nm: $k_{gx}{}^{\left(1\right)}$	465 nm:$\;k_{gx45}{{}^{\left(2\right)}}_{back}$ 595 nm:$\;k_{gx}{{}^{\left(2\right)}}_{back}$

**Table 2:** Preparation and fluorescence characteristics of particle.

Fluorophores	Concentration Prepared	Peak Wavelength/nm(Ex/Em)	Fluorescence Quantum Yield/%
Yellow–green (YG)	2.0 × 10^13^ particles/mL	505/515	44 ^(1)^
Cy5-Sreptavidin (Cy5-SA)	32 nM	650/670	27 ^(2)^
Qdot655-Streptavidin (Qdot655-SA)	5 nM	-/655	80 ^(3)^

^(1)^ Thermo fisher F8787 Certificate of Analysis for Lot 1173470, ^(2)^ Jack Immuno Research Certificate of Analysis for Lot 138512, and ^(3)^ Q10123MP Certificate of Analysis for Lot 1622955.

**Table 3:** The wavelength ranges of filters.

Filter	Excitation Wavelength Range/nm	Emission Wavelength Range/nm
CFP	exCFP: 425–445	―
GFP	exGFP: 460–480	emGFP: 495–540
Cy3	exCy3: 510–555	emCy3: 570–615
Cy5	exCy5: 600–650	emCy5: 670–715

**Table 4:** Enhancement effect of YG with exCFP for the 1D chip in the epi-optical system.

Emission Filter (Wavelength Range)		emGFP (495–540 nm)	emCy3 (570–615 nm)
Total enhancement factor, excitation enhancement and emission enhancement factors (plasmon mode assigned)	Fluorescence spectra	1.4 Ex: 1.4 ($k_{gx}{}^{\left(2\right)}$) Em: 1 (none)	1.4 Ex: 1.4 ($k_{gx}{}^{\left(2\right)}$) Em: 1 (none)
	Fluorescence image	1.4 Ex: 1.4 ($k_{gx}{}^{\left(2\right)}$) Em: 1 (none)	1.4 Ex: 1.4 ($k_{gx}{}^{\left(2\right)}$) Em: 1 (none)

**Table 5:** Enhancement effect of YG with exCFP for the 1D chip in the transmitted-light system.

Emission Filter (Wavelength Range)		emGFP (495–540 nm)	emCy3 (570–615 nm)
Total enhancement factor, excitation enhancement and emission enhancement factors (plasmon mode assigned)	Fluorescence spectra	2.5 Ex: 2.5 ($k_{gx}{}^{\left(2\right)},\;k_{gx}{{}^{\left(2\right)}}_{back}$) Em: 1 (none)	2.2 Ex: 2.2 ($k_{gx}{}^{\left(2\right)},\;k_{gx}{{}^{\left(2\right)}}_{back}$) Em: 1 (none)
	Fluorescence image	2.0 Ex: 2.0 ($k_{gx}{}^{\left(2\right)}$, $\;k_{gx}{{}^{\left(2\right)}}_{back}$) Em: 1 (none)	2.0 Ex: 2.0 ($k_{gx}{}^{\left(2\right)},\;k_{gx}{{}^{\left(2\right)}}_{back}$) Em: 1 (none)

**Table 6:** Enhancement effect of YG with exCFP for the 2D chip in the epi-optical system.

Emission Filter (Wavelength Range)		emGFP (495–540 nm)	emCy3 (570–615 nm)
Total enhancement factor, excitation enhancement and emission enhancement factors (plasmon mode assigned)	Fluorescence spectra	2.0 Ex: 1.3 ($k_{gx}{}^{\left(2\right)}$) Em: 1.5($k_{gx45}{}^{\left(2\right)}$)	1.3 Ex: 1.3 ($k_{gx}{}^{\left(2\right)}$) Em: 1 (none)
	Fluorescence image	2.4 Ex: 1.3 ($k_{gx}{}^{\left(2\right)}$) Em: 1.8 ($k_{gx45}{}^{\left(2\right)}$)	1.3 Ex: 1.3 ($k_{gx}{}^{\left(2\right)}$) Em: 1 (none)

**Table 7:** Enhancement effect of YG with exCFP for the 2D chip in the transmitted optical system.

Emission Filter (Wavelength Range)		emGFP (495–540 nm)	emCy3 (570–615 nm)
Total enhancement factor, excitation enhancement and emission enhancement factors (plasmon mode assigned)	Fluorescence spectra	2.8 Ex: 2.1 ($k_{gx}{}^{\left(2\right)},\;k_{gx}{{}^{\left(2\right)}}_{back}$) Em: 1.3 ($k_{gx45}{}^{\left(2\right)}$)	2.1 Ex: 2.1 ($k_{gx}{}^{\left(2\right)}$, $k_{gx}{{}^{\left(2\right)}}_{back}$) Em: 1 (none)
	Fluorescence image	3.4 Ex: 2.2 ($k_{gx}{}^{\left(2\right)}$, $k_{gx}{{}^{\left(2\right)}}_{back}$) Em: 1.5 ($k_{gx45}{}^{\left(2\right)}$)	2.2 Ex: 2.2 ($k_{gx}{}^{\left(2\right)}$, $k_{gx}{{}^{\left(2\right)}}_{back}$) Em: 1 (none)

**Table 8:** Fluorescence enhancement factors of the fluorophores with emCy5 for the 1D chip in the epi-optical system. The values inside [%] correspond to coefficient variation (CV).

Excitation Wavelength	Excitation Filter (Wavelength Range) Fluorophores	exCFP (425–445 nm)	exGFP (460–480 nm)	exCy3 (510–555 nm)	exCy5 (600–650 nm)
Total enhancement factor, excitation enhancement and emission enhancement factors (plasmon mode assigned)	YG	―	3.1 [6%] Ex: 1 (none) Em: 3.0 ($k_{gx}{}^{\left(1\right)}$)	3.2 [10%] Ex: 1 (none) Em: 3.2 ($k_{gx}{}^{\left(1\right)}$)	―
	Cy5-SA	―	―	5.6 [7%] Ex:1 (none) Em: 5.6 ($k_{gx}{}^{\left(1\right)}$)	5.3 [5%] Ex:1 (none) Em: 5.3 ($k_{gx}{}^{\left(1\right)}$)
	Qdot655-SA	2.5 [10%] Ex: 1.2 ($k_{gx}{}^{\left(2\right)}$) Em: 2.1 ($k_{gx}{}^{\left(1\right)}$)	2.2 [5%] Ex: 1 (none) Em: 2.2 ($k_{gx}{}^{\left(1\right)}$)	2.1 [5%] Ex: 1 (none) Em: 2.1 ($k_{gx}{}^{\left(1\right)}$)	2.2 [9%] Ex: 1 (none) Em: 2.2 ($k_{gx}{}^{\left(1\right)}$)

**Table 9:** Fluorescence enhancement factors of the fluorophores with emCy5 for the 2D chip in the epi-optical system. The values inside [%] correspond to CV.

	Excitation Filter (Wavelength Range) Fluorophores	exCFP (425–445 nm)	exGFP (460–480 nm)	exCy3 (510–555 nm)	exCy5 (600–650 nm)
Total enhancement factor, excitation enhancement and emission enhancement factors (plasmon mode assigned)	YG	―	4.0 [4%] Ex: 1 (none) Em: 4.0 ($k_{gx}{}^{\left(1\right)}$)	5.2 [1%] Ex: 1.3 ($k_{gx45}{}^{\left(2\right)}$) Em: 4.0 ($k_{gx}{}^{\left(1\right)}$)	―
	Cy5-SA	―	―	9.7 [4%] Ex: 1.3 ($k_{gx45}{}^{\left(2\right)}$) Em: 7.5 ($k_{gx}{}^{\left(1\right)}$)	7.5 [5%] Ex: 1 (none) Em: 7.5 ($k_{gx}{}^{\left(1\right)}$)
	Qdot655-SA	3.0 [10%] Ex: 1.1 ($k_{gx}{}^{\left(2\right)}$) Em: 2.8 ($k_{gx}{}^{\left(1\right)}$)	2.8 [10%] Ex: 1 (none) Em: 2.8 ($k_{gx}{}^{\left(1\right)}$)	3.7 [6%] Ex: 1.3 ($k_{gx45}{}^{\left(2\right)}$) Em: 2.8($k_{gx}{}^{\left(1\right)}$)	3.0 [10%] Ex: 1 (none) Em: 3.0 ($k_{gx}{}^{\left(1\right)}$)
